# Propofol: neuroprotection in an *in vitro *model of traumatic brain injury

**DOI:** 10.1186/cc7795

**Published:** 2009-04-27

**Authors:** Jan Rossaint, Rolf Rossaint, Joachim Weis, Michael Fries, Steffen Rex, Mark Coburn

**Affiliations:** 1 Department of Anesthesiology, RWTH Aachen University Hospital, Pauwelsstraße 30, 52074 Aachen, Germany; 2 Institute of Neuropathology, University Hospital of the RWTH Aachen, Pauwelsstraße 30, 52074 Aachen, Germany; 3 Department of Surgical Intensive Care, University Hospital of the RWTH Aachen, Pauwelsstraße 30, 52074 Aachen, Germany

## Abstract

**Introduction:**

The anaesthetic agent propofol (2,6-diisopropylphenol) has been shown to be an effective neuroprotective agent in different *in vitro *models of brain injury induced by oxygen and glucose deprivation. We examined its neuroprotective properties in an *in vitro *model of traumatic brain injury.

**Methods:**

In this controlled laboratory study organotypic hippocampal brain-slice cultures were gained from six- to eight-day-old mice pups. After 14 days in culture, hippocampal brain slices were subjected to a focal mechanical trauma and subsequently treated with different molar concentrations of propofol under both normo- and hypothermic conditions. After 72 hours of incubation, tissue injury assessment was performed using propidium iodide (PI), a staining agent that becomes fluorescent only when it enters damaged cells via perforated cell membranes. Inside the cell, PI forms a fluorescent complex with nuclear DNA.

**Results:**

A dose-dependent reduction of both total and secondary tissue injury could be observed in the presence of propofol under both normo- and hypothermic conditions. This effect was further amplified when the slices were incubated at 32°C after trauma.

**Conclusions:**

When used in combination, the dose-dependent neuroprotective effect of propofol is additive to the neuroprotective effect of hypothermia in an *in vitro *model of traumatic brain injury.

## Introduction

Traumatic brain injury (TBI) is a common consequence of traffic-related accidents and incidents at work and at home. The annual incidence of TBI in the UK is estimated to be approximately 400 per 100,000 patients per year [1]. The treatment of patients with traumatic injury to the brain accounts for a considerable proportion of the budget spent annually on health care and the subsequent costs for rehabilitation, post-hospital long-term care and disability are a significant burden for the economy and society. It should be noted that all currently available therapy approaches for TBI are symptomatic in nature. To date, no clinically established therapy exists that specifically counteracts the actual pathological mechanisms leading to traumatic brain tissue injury.

Propofol (2,6-diisopropylphenol) is a short-acting, intravenous hypnotic agent widely used for the induction and maintenance of general anaesthesia in the perioperative setting, for sedation in intensive care unit patients and for short-time interventional procedures. Propofol has been shown to be an effective neuroprotective agent in certain *in vitro *models of brain injury induced by oxygen-glucose deprivation. To this point, the effects of propofol on the outcome of mechanically induced brain injury have not been investigated.

We demonstrate that the anaesthetic agent propofol (2,6-diisopropylphenol) exerts a strong neuroprotective effect in an *in vitro *model of TBI and that this effect is further amplified when propofol is applied under hypothermic conditions.

## Materials and methods

### Organotypic hippocampal slice cultures

All experiments were performed in compliance with the local institutional Ethical Review Committee and have been approved by the animal protection representative at the Institute of Animal Research at the RWTH Aachen University Hospital, according to the German animal protection law §4, Section 3. Unless otherwise stated, all chemicals were obtained from PAA Laboratories GmbH (Pasching, Austria). The organotypic hippocampal slice cultures were prepared from the brains of six to eight-day-old C57/BL6 mice pups (Charles River Laboratories, Sulzfeld, Germany) as previously reported [2], with some modifications. Immediately after extraction, the brain was submerged into ice cold preparation medium consisting of Gey's balanced salt solution (Sigma Aldrich, Munich, Germany) containing 5 mg/ml D-(+)-glucose (Roth, Karlsruhe, Germany) and 0.1% antibiotic/antimycotic solution (containing penicillin G 10,000 units/ml, streptomycin sulfate 10 mg/ml and amphotericin B 25 μg/ml).

The hippocampi were dissected under stereomicroscopic supervision, placed on a McIllwain tissue chopper (The Mickle Laboratory Engineering Co. Ltd., Gomshall, UK) and cut into 400 μM thick slices. The slices were then transferred into the ice cold preparation medium, separated from each other and placed onto the membrane of a tissue culture insert (MilliCell-CM, Millipore Corporation, Billerica, MA, USA) that was positioned inside a 35 mm tissue culture plate (Sarstedt, Newton, MA, USA). Growth medium containing 50% Eagle minimal essential medium with Earle's salts, 25% Hank's balanced salt solution, 25% heat inactivated horse serum, 2 mM L-glutamine, 5 mg/ml D-glucose, 1% antibiotic/antimycotic solution and 50 mM 4-(2-hydroxyethyl)-1-piperazineethanesulfonic acid (HEPES) buffer solution (Fluka, Buchs, Switzerland) was placed underneath the membrane allowing for substrate diffusion. The culture plates containing the membrane inserts with hippocampal slices on top were incubated at 37°C in a humidified atmosphere of 95% air and 5% carbon dioxide. The growth medium was exchanged 24 hours after preparation and every third day thereafter.

### Traumatic brain injury

After cultivation over a 14-day period the growth medium was replaced with experimental medium which differed from the growth medium with the substitution of horse serum with extra Eagle minimal essential medium and the addition of 4.5 μM propidium iodide (PI; Sigma Aldrich, Munich, Germany). After 30 minutes of incubation with PI, baseline fluorescence imaging was performed. The TBI was produced using a specially designed apparatus as previously reported [3]. The construction of the apparatus was based on previously published descriptions [4-6]. Under stereomicroscopic supervision a stylus with a diameter of 1.65 mm was positioned 7 mm above the CA1 region of the hippocampal slices with the aid of a three-axis micromanipulator and was dropped onto the slice with constant and reproducible impact energy of 5.26 μJ. The drop height, being directly proportional to the impact energy, was chosen so that the neuronal tissue was not ruptured or perforated.

### Intervention

After traumatising the slices, the medium was exchanged for experimental medium containing 4.5 μM PI. PI was present at all times until final imaging. The culture plates with the slices were returned to the incubator with an atmosphere of 95% air/5% carbon dioxide at 37°C for 72 hours before final fluorescence imaging. Slices under these conditions were considered to be the control group. For experimental groups, the medium was exchanged after the traumatising procedure with experimental medium containing propofol (97% purity, Sigma Aldrich, Munich, Germany) at concentrations between 10 and 400 μM dissolved in 0.1% dimethyl sulfoxide (Roth, Karlsruhe, Germany). The slices were incubated at temperatures of 37°C or 32°C, for experiments under hypothermic conditions, for 72 hours before final fluorescence imaging.

### Microscopy and staining

PI is a nucleic acid intercalating agent that is membrane-impermeable in vital cells with intact cell membranes. In damaged cells gaps in the cell membrane allow PI to enter the cell forming highly fluorescent complexes with nuclear DNA [7]. PI intercalates in between the DNA double strands with little or no base sequence preference with a stoichiometry of one dye per four to five base pairs. The fluorescent PI/DNA complexes have a peak emission in the red region of the visible light spectrum. After intercalation both the approximate fluorescence excitation maximum and fluorescence emission maximum are shifted to the right from 488 and 590 nm to 535 and 617 nm, respectively.

Fluorescence images were taken with an upright fluorescence microscope (Zeiss Axioplan, Carl Zeiss MicroImaging GmbH, Jena, Germany) equipped with a rhodamine filter and a low-power ×4 objective lens (Zeiss Achroplan 4×/0.10, Carl Zeiss MicroImaging GmbH, Jena, Germany) and captured with a digital camera (SPOT Pursuit 4 MP Slider, Diagnostic Instruments Inc, Sterling Heights, MI, USA). Image acquisition software (MetaVue, Molecular Devices, Sunnyvale, CA, USA) was used for computer-based control of the microscope and to capture the images from the digital camera. To compensate for the changing intensity of the mercury lamp over time, reference fluorescence measurements using a standard fluorescence slide (Fluor-Ref, Omega Optical, Brattleboro, VT, USA) were performed to adjust the exposure time accordingly prior to every imaging session [3].

### Injury quantification

The tissue injury in the slices was measured by pixel-based image analysis. The images taken with the fluorescence microscope were acquired as eight-bit monochrome images, thus every pixel's gray scale value was encoded with a resolution ranging from 0 (black) to 255 (white). ImageJ (National Institutes of Health, Bethesda, MD, USA) was used to plot a gray scale histogram for each image which shows the sum of all pixels sharing the same gray scale value from 0 to 255. Regions with high gray scale values resembled damaged cells in the images of traumatised slices with high PI uptake and high fluorescence light emission. Images of non-traumatised slices showed a sharp, well-defined peak at gray scale values between 20 and 75 (darker background coloured portions of the image) falling rapidly to near zero at gray scale values of over 75.

Using a series of control experiments a threshold of 75 was established above which in non-traumatised slices just low sums of pixels could be found. This method has been used in previous publications [3,5,6]. The histograms of images from traumatised slices showed a lower but broader background signal peak which was slightly shifted to the right and a second, well-defined peak at gray scale values between 160 and 180 (majority of highly fluorescent, damaged cells). The histogram curve beyond a gray scale value of 75 was integrated. The results yielded a profound, quantified measure of the PI fluorescence and thus of the cell injury in the slices. The normalised integral was defined as the trauma intensity. This analysis was performed for each slice in every group. Two types of tissue injury were defined: 'Total injury' as the complete injury over the slice and 'secondary injury' as the injury over slice excluding the primary impact site of the stylus. For the calculation of the secondary injury we created a mask with the same diameter as the stylus using ImageJ. The mask was positioned exactly over the stylus' impact site in the images and excluded this area from the pixel analysis and thus the calculation of the trauma. The same mask was applied to every image when calculating the secondary injury.

### Statistical analysis

Throughout this article, the total and secondary injury are expressed as fractions relative to the total injury observed after 72 hours under control conditions (37°C), which was normalised to unity. For each experimental condition a mean number of 17 slices was used (minimum number = 12, maximum number = 26). The mean value and the standard error of the mean (SEM) were calculated for the trauma intensities of the slices in each group using SPSS software version 16.0 (SPSS Inc., Chicago, IL, USA). The test for statistical significance was also performed with SPSS using an analysis of variance (ANOVA). A *P *≤ 0.05 was taken as statistically significant.

## Results

**Figure 1:**
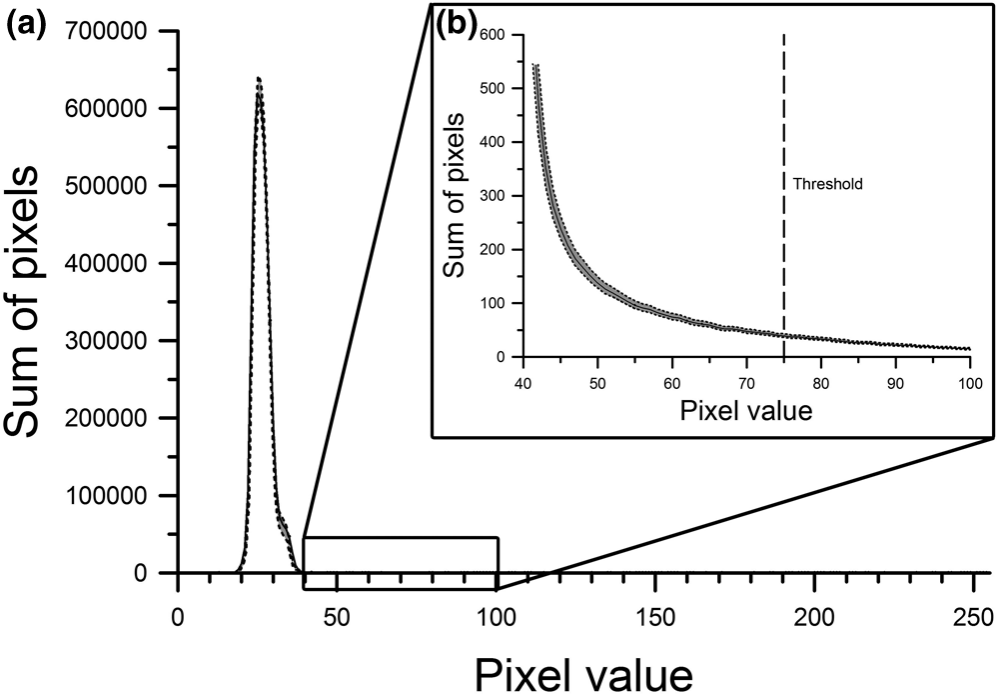
After 30 minutes of incubation with propidium iodide, baseline fluorescence imaging was performed on all slices to qualify the level of cell injury prior to trauma. Histograms were computed from images by counting the sum of all pixels sharing the same eight-bit gray scale value (from 0 to 255). **(a) **The mean with the standard error of the mean (SEM) of a total of 206 slices included in this setting is shown. **(b) **An enlarged portion of the graph around the applied threshold of 75 (dashed line) is shown. The continuous line is the mean value, and the dotted lines are the upper and lower bounds of the SEM. The data demonstrates the continuous low level of injury throughout the slices prior to traumatisation.

A very low level of tissue injury was maintained in all slices prior to inclusion in the study groups. This initial injury could be observed in all slices and is attributable to minimal cell death originating from the preparation procedure and to influential effects regarding the handling and maintenance of the slice cultures over the 14-day cultivation time period. Yet the total trauma signal in the baseline fluorescence measurement was very low when compared with the maximum total trauma signal observed after 72 hours in the trauma control group at 37°C (0.004 ± 0.0004 vs. 1.00 ± 0.14; *P *= 0.00). The level of injury was persistent over all slices with very little variation. This is demonstrated by the data in Figure 1.

**Figure 2:**
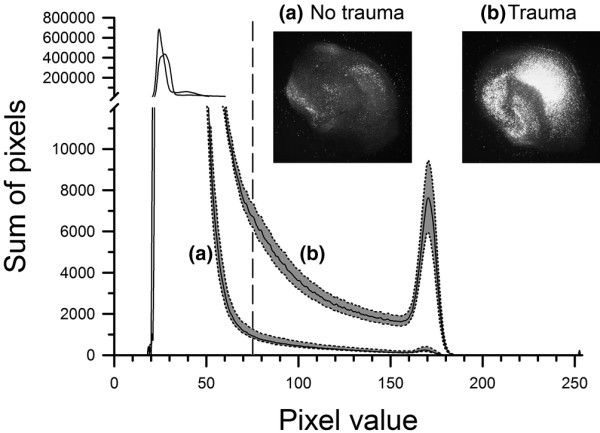
After preparation, cultivation for 14 days and baseline measurement, slices were either traumatised or not by the impact of a stylus onto the CA1 region of the hippocampus. The extent of the trauma was evaluated by fluorescence imaging 72 hours after the induced trauma and pixel-based image analysis. Curve a shows the histogram of non-traumatised slices (n = 17) at t = 72 hours. The straight line is the mean value, the dashed lines are the upper and lower bounds of the standard error of the mean. Curve b shows the histogram of traumatised slices (n = 17). For a better view of the important section the y-axis was split at 12,000 and two different scales were used in both parts. The vertical dashed line is the applied threshold at a gray scale value of 75. The integral of all pixel values greater than the threshold was calculated for each group and defined as the trauma intensity. Two example images for **(a) **non-traumatised and **(b) **traumatised slices at t = 72 hours are shown in the upper right corner.

The next step was to identify the characteristics of traumatised and non-traumatised slices with respect to their histograms in order to establish a quantitative measurement tool to express the extent of the tissue trauma. The comparison between traumatised and non-traumatised control group slices yielded very different trauma signals in the fluorescence microscope image histograms (Figure 2). The traumatised slices showed a reduced peak in background signal between gray scale values of 20 and 50 and a shift towards greater gray scale values with a discrete peak at values between 160 and 180. Through these control experiments a threshold was established at a gray scale value of 75. Therefore, gray scale values greater than 75 can be attributed to the traumatised tissue, which became fluorescent due to PI uptake. The portion of the histogram curves with a gray scale value greater than 75 were integrated and the result used as a direct, quantitative figure for the measurement of the extent of traumatic injury.

**Figure 3:**
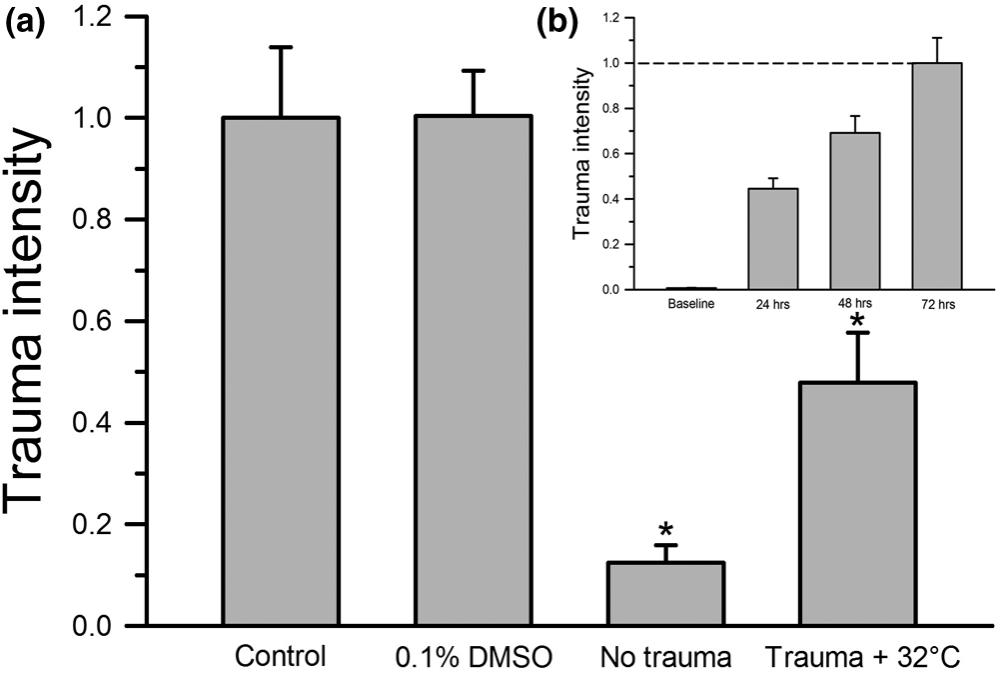
All groups were normalised against the trauma control group at t = 72 hours. **(a) **No significant difference between the untreated traumatised control group and the group treated only with 0.1% dimethyl sulfoxide (DMSO) could be observed. The addition of 0.1% DMSO was necessary to solve the lipophilic propofol in an aqueous medium. The detected trauma in the non-traumatised group and in the group with hypothermia was significantly lower compared with the trauma control group. * *P *≤ 0.05. **(b) **The trauma intensity increased steadily over time, which has been shown before. This is demonstrated by slices (n = 14). Values were normalised against the trauma intensity at t = 72 hours.

The trauma intensity increased steadily over time after trauma. This is demonstrated by the data in Figure 3b in slices that were evaluated for trauma development using fluorescence microscopy at 0 hours (0.006 ± 0.002), 24 hours (0.45 ± 0.05), 48 hours (0.69 ± 0.08) and 72 hours (1.00 ± 0.11) post-traumatisation. This slow development of the injury stands in contrast to the quickly achieved equilibrium state of PI binding to the DNA in damaged cells, which has been proven in previous publications [3], so the observed increase in fluorescence over time can be attributed to the ongoing cell death rather than to delayed PI uptake and fluorescence development. The values in panel 3b were normalised against the trauma intensity at t = 72 hours. The results of these control experiments justify the sole measurement of the trauma after 72 hours and therefore we evaluated the tissue trauma only at this time point.

Dimethyl sulfoxide (DMSO), the solving agent needed to solve the lipophilic propofol in the aqueous medium, had no detectable effect on the total tissue trauma when administered. This is demonstrated by the level of total trauma intensities in the control group and the group treated with 0.1% DMSO (1.00 ± 0.14 vs. 1.00 ± 0.09; Figure 3a). The comparison of the trauma intensities between the trauma control group and the non-traumatised group at 37°C yielded a significant difference (1.00 ± 0.14 vs. 0.13 ± 0.04, *P *= 0.00), as expected.

**Figure 4:**
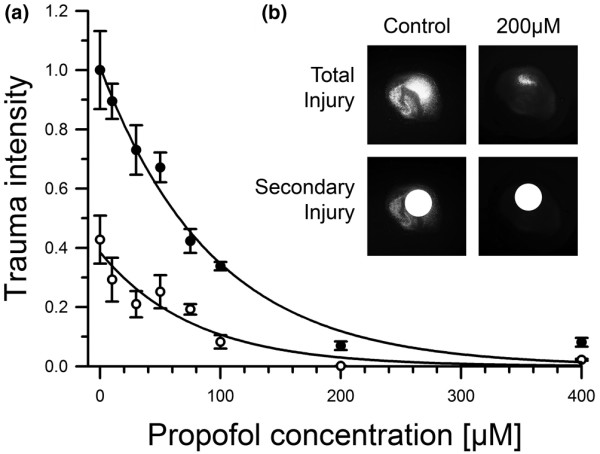
The extent of tissue trauma in the slices was quantified using pixel-based analysis of the acquired fluorescence images at t = 72 hours after trauma. Total injury was defined as the total level of cell death in the slices. A minimum of 12 slices were evaluated per group. Secondary injury was calculated by covering the pin's direct impact site in the images with a defined mask excluding this area from trauma analysis. **(a) **The concentration-response curves of propofol from 10 to 400 μM for both total (filled circles, upper curve) and secondary injury (open circles, lower curve). **(b) **Exemplary images for total and secondary injury (showing the impact site exclusion mask) in the control group and at a propofol concentration of 200 μM. Non-linear regression curves were fitted into the graph to visualise the trends.

Propofol at normothermia (37°C) was added to experimental medium at concentrations of 10, 30, 50, 75, 100, 200 and 400 μM. Figure 4a displays the concentration-response curves for both the total (filled circles, upper curve) and secondary injury (open circles, lower curves). The nonlinear regression curves were fitted into the graph to visualise the trend. Figure 4b shows exemplary fluorescence images for total and secondary injury (with applied mask for exclusion of the stylus' direct impact site) in the trauma control group and the group treated with 200 μM propofol. A clearly visible reduction of fluorescent, dead cells can be observed when comparing the images of slices from each group, for total and secondary injury. The total trauma intensities under normothermic conditions were 0.86 ± 0.13 (10 μM), 0.73 ± 0.06 (30 μM), 0.67 ± 0.08 (50 μM), 0.42 ± 0.04 (75 μM), 0.34 ± 0.05 (100 μM), 0.07 ± 0.01 (200 μM) and 0.08 ± 0.02 (400 μM). The secondary injury intensity in the control group under normothermic conditions was 0.43 ± 0.08. The intensities of the observed secondary trauma with propofol treatment after trauma under normothermic conditions were 0.29 ± 0.07 (10 μM), 0.21 ± 0.05 (30 μM), 0.25 ± 0.06 (50 μM), 0.19 ± 0.02 (75 μM), 0.08 ± 0.02 (100 μM), 0.001 ± 0.0001 (200 μM) and 0.02 ± 0.003 (400 μM).

**Figure 5:**
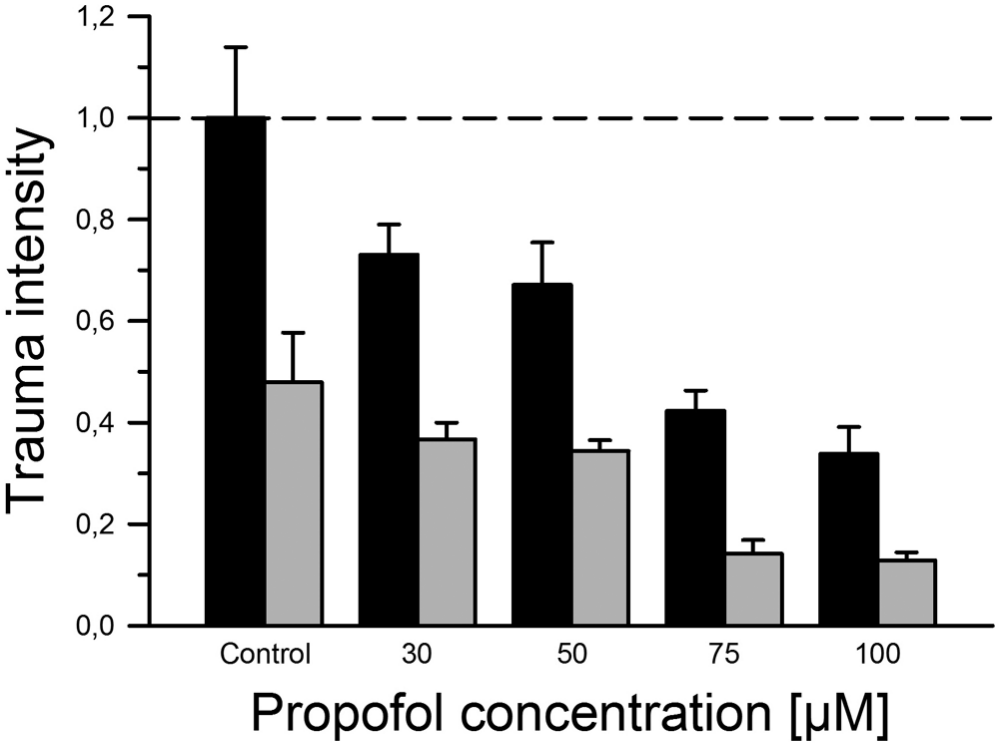
The use of hypothermia at a temperature of 32°C decreased the trauma intensity. All groups were normalised against the trauma control group at 37°C. The black bars resemble the trauma intensity at normothermia (37°C) and propofol concentrations from 0 to 100 μM after t = 72 hours. The grey bars are the corresponding trauma intensities for slices treated with the same concentrations of propofol but kept at hypothermia (32°C) for 72 hours.

Hypothermia at 32°C alone decreased the total tissue trauma in this model by more than 50% (0.48 ± 0.10 vs. 1.00 ± 0.14, *P *= 0.015), similar to previous reports [2-4]. A significant reduction of total traumatic injury in hypothermia groups when compared with the corresponding groups treated with the same concentration of propofol under normothermic conditions could be observed at propofol concentrations of 30 μM (0.37 ± 0.03 vs. 0.73 ± 0.06; *P *= 0.00) 50 μM (0.34 ± 0.02 vs. 0.67 ± 0.08; *P *= 0.00) 75 μM (0.14 ± 0.03 vs. 0.42 ± 0.04; *P *= 0.00) and 100 μM (0.13 ± 0.02 vs. 0.34 ± 0.05; *P *= 0.00), as shown in Figure 5. Thus, the use of mild hypothermia (32°C) in combination with propofol at concentrations between 30 and 100 μM showed a remarkable effect in reducing total tissue trauma. The trauma reduction between hypothermia groups and the corresponding normothermia groups ranged from 48.7% (30 μM) to 66.4% (75 μM) with a mean reduction of 55.7%. The analysis using a two-way ANOVA revealed a statistical significance (*P *< 0.0001) of both factors (propofol concentrations and temperature) when applied independently. The interaction, beyond the additive effect, of the two factors was not statistically significant (*P *= 0.397).

In summary, post-traumatic administration of propofol led to a dose-dependent decrease in total as well as secondary tissue trauma, and the combination of propofol and hypothermia were additive in regard to neuroprotection.

## Discussion

We have investigated the potential beneficial neuroprotective effects of propofol in an *in vitro *setting of TBI utilising the well-established [2,3,5,6,8-17] model of organotypic hippocampal slice cultures. We could show that propofol greatly diminishes both total and secondary injury when administered in our *in vitro *model of TBI. A dose-dependent neuroprotective effect can be observed in the concentration range between 10 and 400 μM propofol (Figure 4).

This method yields easy and open access to the nervous tissue *in vitro *for manipulation and assessment. Yet, in contrast to dissociated cell cultures, it retains most of the features of tissue organisation as a heterogeneous population of cerebral cells and functional characteristics, e.g. the preservation of synaptic and anatomical organisation, with great similarities to the *in vivo *state [2,8,11,13,14,16,18-21]. Hence, organotypic hippocampal slice cultures are an appropriate compromise between models using dissociated cell cultures and experimental *in vivo *models with whole living animals [4,13,22,23]. The method of selectively traumatising the hippocampal slices has been widely described and used before [3-6,22]. To a certain extent, it shares *in vitro *the characteristics of cerebral traumatic injury *in vivo*. We selectively traumatised the vulnerable CA1 region of the hippocampus. The subsequent occurrence of focal injury at the primary site of impact and the development of secondary injury distant to that site are also comparable with the *in vivo *situation. Thus, our model can be used as a testing environment for experimental treatments with a sufficiently high level of confidence.

The development of post-traumatic and post-ischaemic secondary injury has been analysed using this model in previous studies [3,4]. A number of possible molecular and cellular causes including the activation of pro-apoptotic mediator pathways [24-26], up-regulation of cell death genes [12], free radical generation, excitotoxicity [8] and cell-to-cell electrical communication possibly involving gap-junctions [21] have been identified. In fact, observations have been made that secondary injury following TBI shares similarities with the post-ischaemic neuronal damage observed in the penumbra surrounding an ischaemic core following stroke [27]. This may give rise to the assumption that similar neuroprotective strategies may be successful in both aetiologies of brain injury.

PI was used in this method as a staining agent to assess the degree of tissue injury. PI labelling has been proven to correlate well with the extent of neuronal injury [7,17,28] and used as a cell viability marker in previous studies [3-5,9,14,17,29]. It is generally accepted that there is a linear correlation between the cumulative fluorescence emission in PI-treated tissue and the number of damaged cells when compared with cell viability assessment relying on morphological criteria [4,14,17].

The exact pharmacodynamic mechanism of action of propofol is not fully known as yet. However, evidence indicates that it primarily acts by potentiating the function of the gamma-aminobutyric acid (GABA)_A _and possibly glycine-receptors [30-32]. Additionally, recent results suggest that propofol may interact with the endocannabinoid system [33,34], which could contribute to its anaesthetic properties. Propofol has previously been under investigation in both *in vivo *and *in vitro *models of ischaemia-reperfusion injury and oxygen-glucose deprivation. The *in vitro *studies have yielded both positive [9,35-38] and negative [10,15,39] results for the neuroprotective benefits of propofol. In various *in vivo *studies a neuroprotective effect in terms of post-ischaemic cerebral damage reduction could be demonstrated in models of transient [40-44] but not permanent [45] focal ischaemia. The effects of propofol in mechanical TBI have rarely been investigated to date, although there is evidence that propofol can protect neurons from acute mechanically induced cell death following dendrotomy by potentiation of GABA_A_-receptor functions [46].

Two *in vivo *studies in rodent models yielded negative results concerning the neuroprotective effect of propofol [47,48]. The results of these studies are in contrast to the findings presented in this study. This may be due to different circumstances found in experimental models using whole living animals with all present systemic variables, which are absent in our model of TBI. In addition, the six-hour period of post-traumatic propofol application until the final assessment of brain injury was significantly shorter than the three-day period used in our model. There is also a difference in the propofol concentrations used and the point of application, which is beyond the blood-brain barrier in our study. Recent studies have focused on the concentrations of propofol in the blood serum, cerebrospinal fluid and brain parenchyma and found that when propofol is administered directly via the nutrient medium in a model of organotypic brain slices a final equilibrium concentration of propofol in the brain parenchyma is not reached until 360 minutes after the start of propofol administration [49].

Hypothermia at 32°C alone had a strong effect in reducing the trauma intensity by more than 50% (Figure 3). These results are not entirely surprising because the neuroprotective benefit of hypothermia has been demonstrated before [2-4,50]. When hypothermia was combined with propofol at concentrations between 30 and 100 μM, a further reduction in total traumatic injury could be achieved (Figure 5).

There are several issues in this study that must be clarified. First, the maximum clinically feasible propofol concentration in the cerebral tissue remains unclear. Some authors consider the concentrations used in this study (10 to 400 μM) to be reasonable [9,10,51-54], whereas they are considered to be too high by others [49,55,56]. Second, the hippocampal slice culture model, besides its many favourable advantages, bears certain disadvantages that need to be mentioned. Due to the nature of the model it excludes mechanisms of injury that may influence brain damage in the *in vivo *situation such as the absence of any injury pathways related or due to brain swelling inside an enclosed skull, reperfusion injury, global or local ischaemia and/or hypoxia and other systemic variables. Third, propofol was administered directly following the traumatisation procedure excluding the effects of a delay possibly encountered in clinical routine management of patients with TBI.

When interpreting our results with attention towards the scientific value and its importance for possible future medical application one should consider the positive findings made are based on a simplified *in vitro *model of TBI similar but still distant from the situation in the patients seen and treated every day. Still, our results possibly contribute to the development of alternative treatment options for TBI and encourage further research in that field, preferably in studies involving whole living animals.

## Conclusions

In this study we could show that propofol is an effective neuroprotective agent when administered after TBI in the hippocampal slice culture model. Propofol reduced both the total tissue injury as well as the secondary injury distant to the primary site of brain injury. This effect was dose-dependent and increased up to 400 μM, the greatest concentration of propofol that was tested. Hypothermia at 32°C alone reduced the tissue injury by about factor two. When hypothermia and propofol were combined a cumulative effect could be observed and the extent of brain injury was further reduced throughout all concentrations that underwent investigation.

## Key messages

• We found that propofol exerts a neuroprotective effect when administered after TBI in this model of organotypic hippocampal slice cultures.

• We could establish a dose-response relationship showing a decrease in neuronal cell death with increasing concentrations of propofol.

• The use of hypothermia at 32°C alone after TBI reduced the extent of neuronal cell death by about factor two.

• There was an additive neuroprotective effect of propofol in combination with hypothermia at 32°C.

## Abbreviations

ANOVA: analysis of variance; DMSO: dimethyl sulfoxide; GABA: gamma-aminobutyric acid; HEPES: 4-(2-hydroxyethyl)-1-piperazineethanesulfonic acid; PI: propidium iodide; SEM: standard error of the mean; TBI: traumatic brain injury.

## Competing interests

The authors declare that they have no competing interests.

## Authors' contributions

JR conducted the experimental laboratory work, performed the statistical analysis and drafted the manuscript. RR participated in the study design and coordination and helped to draft the manuscript. JW, MF and SR helped to draft the manuscript. MC conceived of the study, participated in the study design and coordination and helped to draft the manuscript. All authors read and approved the final manuscript.
